# Orbital apex syndrome associated with intraorbital metastasis of lung cancer

**DOI:** 10.1002/rcr2.922

**Published:** 2022-02-28

**Authors:** Takashi Ookuma, Ryota Kikuchi, Hiroyuki Takoi, Kazutoshi Toriyama, Shinji Abe

**Affiliations:** ^1^ Department of Respiratory Medicine Tokyo Medical University Hospital Tokyo Japan

**Keywords:** lung cancer, ocular motility disorder, optic nerve disorder, orbital apex syndrome, ptosis

## Abstract

In lung cancer patients, brain metastasis is often suspected when neurological symptoms appear; however, if ptosis, ocular motility disorder or optic nerve disorder is observed, it is important to identify and treat the orbital apex syndrome.

We present the case of a patient with lung adenocarcinoma stage IVB diagnosed as orbital apex syndrome (OAS) associated with intraorbital metastasis of lung cancer. When patients with lung cancer have diplopia, ptosis or ocular motility disorder, identifying OAS is important.

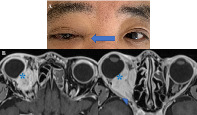

## CLINICAL IMAGE

**FIGURE 1:**
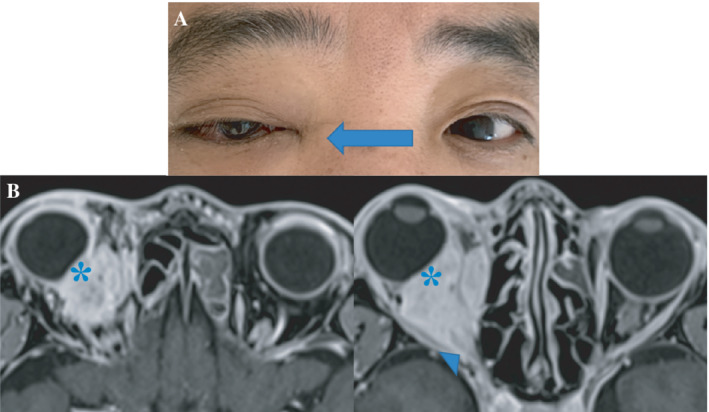
(A) Right eye ptosis and right eye abduction limitation. (B) The orbital magnetic resonance image showed mass in the right orbit excluded the right eye (asterisk) and extended to the apex of the orbit (arrowhead)

A 53‐year‐old male patient with lung adenocarcinoma stage IVB complained of decreased right vision and diplopia when he was hospitalized to begin chemotherapy. His right eyeball was protruding and right eyelid was drooping (Figure 1A). Neurological examination revealed adduction, abduction (Figure 1A) and vertical restriction of the right eye movement. A magnetic resonance image (MRI) revealed a mass in the right orbit that excluded the eye, extending to the orbit's apex (Figure 1B). Clinically, the patient was diagnosed with orbital apex syndrome (OAS) associated with intraorbital metastasis of lung cancer. He was treated with carboplatin, pemetrexed and pembrolizumab, and his right eye vision loss, ptosis and ocular motility disorder partially improved. OAS has a predominant lesion in the superior orbital fissure and optic canal. It presents with III, IV, V1 and VI cranial nerve and optic nerve damage.^1^ Causes include infection, trauma, thrombosis, vasculitis and tumours.^2^ MRI is the preferred diagnostic imaging method for evaluating patients with OAS.^1^ Treatments include therapies for the underlying disease. Lesions in the orbital tip have a profound effect on visual function because of the collection of all eye‐related nerves. When patients with lung cancer have diplopia, ptosis or ocular motility disorder, identifying OAS is important.

## CONFLICT OF INTEREST

None declared.

## AUTHOR CONTRIBUTION

Takashi Ookuma and Ryota Kikuchi designed the research; Shinji Abe, Ryota Kikuchi, Hiroyuki Takoi and Takashi Ookuma analysed the data; and Takashi Ookuma and Ryota Kikuchi wrote the paper.

## ETHICS STATEMENT

The authors declare that appropriate written informed consent was obtained for the publication of this manuscript and accompanying images.

## Data Availability

The data that support the findings of this study are available from the corresponding author upon reasonable request.
